# The Role of China in the UK Relative Imports from Three Selected Trading Regions: The Case of Textile Raw Material Industry

**DOI:** 10.3390/ijerph14121481

**Published:** 2017-11-30

**Authors:** Junqian Xu

**Affiliations:** School of Business, Jiangnan University, Wuxi 214122, China; jxu2000@jiangnan.edu.cn; Tel.: +86-510-8519-7392

**Keywords:** textile materials, UK, China, competitiveness index, comparative advantages

## Abstract

The UK textile industry was very prosperous in the past but in the 1970s Britain started to import textile materials from abroad. Since 1990, half of its textile materials have been imported from the EEA (European Economic Area), ASEAN (Association of Southeast Asian Nations) and North America countries. Meanwhile, UK imports from China have increased dramatically. Through comparisons, this paper calculates the trade competitiveness index and relative competitive advantages of regions and investigates the impact of Chinese textiles on UK imports from three key free trade regions across the textile sectors in the period 1990–2016 on the basis of United Nation Comtrade Rev. 3. We find that China’s textile prices, product techniques, political trade barriers and even tax system have made a varied impact on the UK’s imports across related sectors in the context of green trade and the strengthening of barriers, which helps us recognize China’s competitiveness in international trading and also provides advice on China’s sustainable development of textile exports.

## 1. Introduction

The UK, as the oldest developed country whose textile industry used to be leading in the world, domestic raw textile production can satisfy the needs of its whole textile industry. But from the 1990s, due to the increase in labor costs, the structure British manufacturing industry shifted and transformed, the UK has become a raw textile import country. Other contributory factors to the shift from the domestic market to the developing countries were the lack of domestic production factors and continued demand in the UK for labor-intensive raw materials for textiles. At present, China has become one of the most important countries for the UK and European countries as a source of textile related materials [1]. There are lots of analysis in the field of trade at both aggregate and sector levels [2,3,4,5], but few studies have paid attention to the UK relative imports from others in relation to China from the perspective of textile raw materials while China has become the largest raw textile exporter in the world in the early 1990s and has also significantly affected textile industry in both developed and developing countries [6]. we would like to analyze the UK raw textile imports from representative trading regions rather than specific country in the international market in this study and since 50% of the UK’s total imports of this kind were from European Economic Area (EEA), Association of Southeast Asian Nations (ASEAN) and North America, these three major trading regions will be considered to investigate the role of China on the UK raw textile imports in international market. 

As a high polluting industry whether raw materials or finished products are concerned, the harmful substances in the processing and finishing stages for textiles always affect the environment and public health. Therefore, the EEA has consistently adopted green trade barriers, which at the same time have protected the domestic textile industry. On the one hand, it prevents developing countries from dumping textiles through strict examination of the quality of imports; on the other, prevents domestic consumer health and the environment from being polluted by imports. In their production structure, the ASEAN countries have characteristics similar to China’s, such as having the comparative advantages of labor and price. Meanwhile, in terms of the volume of textile products, the North America free trade region has special advantages for its exports. The product of cotton in the U.S. was number one in the world and Mexico has signed 12 free trade agreements with 44 countries, which have led to zero customs duties when exported to the European Union.

From 1990 to the present, UK textile imports from the EEA, ASEAN and North America have consistently formed 50% of all raw textile imports (Table 1). As a member of EEA, the UK imported raw textiles mainly from the EEA countries, far more than it imported from China and other regions. Though UK imports from the EEA declined after 2000, it still has overwhelming advantages compared with other regions. Besides those from the EEA, UK imported textiles from ASEAN persistently remained at 2% of total imports. The share slightly increased in 2000 but fell back to 2% afterwards. UK imports from North America were larger than those from ASEAN, but tended to decrease after 2000.

Even though EEA and ASEAN countries also have obvious comparative advantages in raw textile, UK imports from China changed from 2% in 1990 to 20% in 2016. This paper further investigates the impact of dramatically increased UK imports of Chinese textile raw materials on its imports from the three large trade regions, the EEA, ASEAN and North America by comparisons and will suggest countermeasures whereby Chinese raw materials can maintain their international competitiveness and sustainable development. 

The study includes other sections as follows, section two introduces the UK textile raw materials imports from three large trade regions, section three is the modeling and data description, section four briefly describe the econometric modeling used in this empirical study, section five draws some conclusions.

## 2. UK Textile Raw Materials Imports

### 2.1. Comparisons between China and Three Free Trade Regions in Competitiveness and Revealed Comparative Advantages

In the 1970s and 1980s, the British textile industry under market pressure exposed problems of lack of production that resulted from engaging in fabric alone. At the same time, due to the impact of other emerging industries and excessive minimum wages, etc., the increasing costs of textile production caused a significant loss of textile workers’ jobs and a great decline in production volume [1]. Subsequently, the UK textile industry began a design-oriented transformation from pure raw materials production [7,8]. The UK has steadily turned into only an importer of textile materials [9].

**Figure 1:**
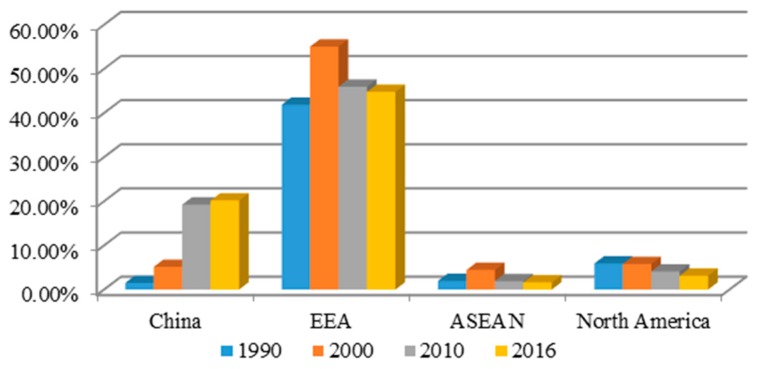
UK textile materials imports from regions as a percentage of total textile imports (%). Source: UN ComTrade SITC Rev. 3, 2017.

As shown in Table 1, UK textile materials imports from China and three free trade regions took up 70% of total imports in textiles. Figure 1 shows the shares in the UK textile materials imports from these regions every ten years. Under EEA trade protection rules, the UK textile imports came mainly from the EEA region. The proportion of UK imports from China increased after 2000, but from other EEA, ASEAN and North America tended to decrease, though these regions still enjoyed comparative advantages.

Trade competition indices in the UK market in textile materials for these three trade regions and China are shown in Table 2. The EEA competition index ranged from 0.255 to 0.277 and there was a decreasing trend after 2016. Imports from China were lower than those from the EEA, but China’s textile competition index increased from twice as high as the EEA to more than 3 times as high. Compared with China, though ASEAN has lower cost advantages, its competition index has steadily fallen and even become negative. In the North American region, even though the UK’s textile imports were larger than those from ASEAN, its competition index was still negative. Table 2 indicates that China’s textile materials in UK markets had incomparable competition advantages and will beyond doubt have great influence on those in other trade regions.

Regarding textile imports in total, Table 3 shows the revealed comparative advantages (RCA) in the UK market of China and the three trade regions. In 2000, the RCA of these three trade regions were all larger than that of China. The RCA of China was stable and kept within the range of 0.624–0.626, but the RCA of the three regions started to decline, which indicates that comparative advantages of Chinese textile materials was becoming relatively strong.

### 2.2. UK Textile Materials Imports from EEA 

Until 2017, the EEA had 31 member countries, including the UK. Table 4 shows that the UK’s textile imports from these EEA members generally declined. Imports from some countries, such as Bulgaria, Hungary, Latvia, Lithuania, Poland, Holland, Romania, Spain, Sweden and Slovakia, increased with small magnitude changes. EEA trade protection rules made the UK favor these countries exports, especially the textile materials from France, Germany, Greece, Ireland, Italy, Holland, Portugal and Spain, all agriculturally developed countries. The textile materials of these regions have comparative advantages among UK imports [10,11].

Table 4 shows that UK textile imports from EEA in 2000 were generally greater than those after 2000. Even though the UK still favors textile materials from these EEA members and 13 new members joined the EEA after 2000, the UK’s import shares from the EEA consistently declined.

### 2.3. UK Textile Materials Imports from ASEAN

As the cost of resources and labor in China increased in general, the advantageous status of Chinese textile materials in the international market lost ground. The ASEAN countries have similar development and production factors to China’s, are highly competitive rivals of China and also rely on the China market. As Table 1 shows, UK textile imports from ASEAN in the 1990s were above those from China but started to shrink due to the financial crisis. Table 5 shows that the UK textile raw materials imports from ASEAN come mainly from Malaysia, Indonesia, Thailand and Vietnam, all labor-intensive industries and countries with extensive supplies of labors, the demand for imports from ASEAN tended to decline steadily after 2000, when China joined the WTO.

### 2.4. UK Textile Materials Imports from North America

The U.S., Canada and Mexico founded the North America free trade region in 1992, as the largest free trade region in the world and also one of the most important sources of cotton and fabric imports to the UK. As shown in Table 6, the quality of U.S. cotton is stable and has strong market competitiveness because of its prompt delivery. Mexico is also one of the world’s leading textile traders and also one of the strongest competitors of China textile industry in the world [12].

UK imports from North America came mainly from the U.S., but showed a declining trended after 2000, whereas the demand for imports from Mexico suddenly increased after 2010, though they fell after that year. Generally speaking, the UK’s import demand from North America did not increase significantly after 2000, like its demand of such imports from the EEA and ASEAN.

### 2.5. Status of China in the UK’s Textile Materials Imports

According to the HM (Her Majesty) Revenue and Customs statistics of the UK in 2016, bilateral trade between the UK and China was valued at 18.95 billion dollars and the value of UK imports was 14.49 billion dollars, accounting for 9.6% of the UK’s imports in total. The UK deficit was 10.02 billion dollars. Of all imports, textiles and materials was the third largest category of UK import products, taking up 13.4% of the total. In terms of textile trading, Chinese labor-intensive products still had leading comparative advantages in the world market and accounted for 23.4% of all UK imports [13].

Both Graph 1 and Table 1 show that UK imports from China significantly increased from 1990 to 2017. We can observe the level of textile materials exports in the period 1990–2016. In the early 1990s, China’s textile raw materials were at a low ebb, subjected to quota restrictions. Since the middle of 1970s, trade protectionism in Europe and the U.S. has become more and more serious and a series of export restriction policies has even been launched to cope with textile exports from developing countries [14,15,16]. An international textile trade arrangement signed in 1974, the Multifiber Arrangement (MFA), regulated that textile importers should begin discriminatory quantity control on developing textile exporters. When the MFA was replaced by the Agreemnt on Textiles and Clothing (ATC) in 1995, China became the biggest textile exporter in the world and textile raw materials started to rise dramatically, especially after China gained membership of the World Trade Organization (2000) [17]. 

After 2000, textile trade policy became more relaxed for China but friction over textile exports still arose consistently [18,19]. From the international perspective, some developed countries set up a quality certificate system with more and more advanced detection technology and a complicated qualify index. The EU’s trade protection policy is related to environmental protection regulations and textile trade protection policies; it implemented safety and health tests and even put forward restrictions on dyestuffs that lacked environmental protection. The UK strictly implemented these environmental restrictions in its exports [9], which indicate that the stricter the restrictions, the higher requirements of textile imports. Though Chinese textile export-led enterprises have comparative advantages in costs and price in international market, in order to satisfy the requirements of textile import needs of developed countries, Chinese textile industry also need to make strict restrictions on regulations in the process of textile raw materials selection and textile production on the bases of international standards, the enterprises that cannot fit these changes gradually will lose its market share and cannot survive in the long run as a result. Consequently, the trade barriers have increased the adaptive costs of textile export-led enterprises, and price of textile materials and products, which have affected the competitiveness of Chinese textile firms in international textile market [12].

**Figure 2:**
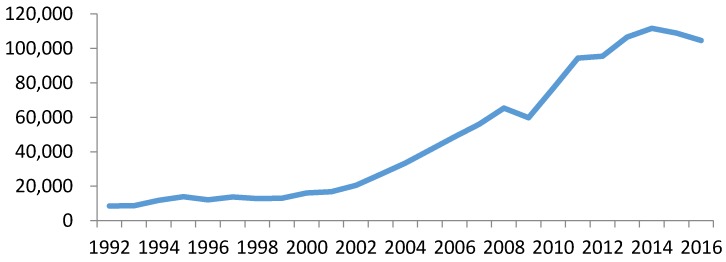
Value of Chinese total exports (unit: dollars in millions). Source: UN ComTrade SITC Rev. 3, 2017.

**Figure 3:**
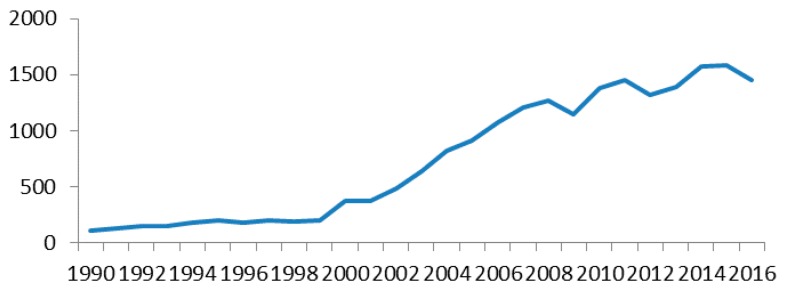
UK textile imports value from China (unit: dollars in millions). Source: UN ComTrade SITC Rev. 3, 2017.

Under the pressure of international trade competition, China stopped paying customs duties on 17 textile materials and products from domestic side, which released the potential of sustainable development for China raw textiles exports; this is confirmed by Figure 2 and Figure 3, which show that Chinese raw materials exports increased dramatically. Figure 3 further indicates that UK textile imports from China increased two-fold after 2005.

The textile industry is an industrial pillar of China. China has incomparable comparative advantages in the international market beyond the EU under protectionism and ASEAN with more comparative advantages. 

## 3. Model

As the largest textile raw material producer, China has obvious comparative advantages in textile exports and the relative price of Chinese textile raw materials may have diverse effects on different sectors of textiles at different times. Bini-Smaghi (1991) [20] suggests that different countries/sectors make different import demands and price elasticities. Broda and Romalis (2004) [21] conducted a bilateral sectoral study and used data separated into “differentiated” and “commodity” trade following Rauch (1999) [22]. Some studies that have examined bilateral sectoral trade using sectoral prices instead of country aggregates include those of Belanger (1992) [23], de Vita and Abbott (2004) [24], Peridy (2003) [25]; Broda and Romalis (2004) [21] and Feenstra (2003) [26] respectively use unit value and industrial production prices to do this. De Vita and Abbott (2004) [24] use the multilateral sectoral export price and Byrne (2006) [27] uses the industrial value added deflator.

The “Theory of Demand for Products Distinguished by Place of Production” (Armington, 1969) [28] assumes that imported goods and their domestic counterparts are incomplete substitutes. Elasticities of substitution among imports and competing domestic production (Armington elasticities) play a key role in open-economy computable general equilibrium (CGE) modeling. Armington elasticities used in CGE models refer to heterogeneous product groups rather than homogeneous products, thus reflecting differences in the composition of imported and domestic production.

At the conceptual level, the Armington framework is an early version of the love-for-variety approach in consumer theory. This suggests that imported and domestic goods differ from each other in the perception of the representative agent, and the Armington elasticity reflects the degree of perceived difference. The Armington model estimates both price and output elasticities and assumes separable consumer utility for goods in an industry from the consumption of other products. A variety of functional forms has been used to model the demand for imports and domestic output by industrial category. Armington (1969) [27] and most CGE modelers have used the constant elasticity of substitution (CES) form for the representative agent’s subutility function for an industry group, where utility is derived from domestic and foreign goods,

(1) $$U={\left[\gamma M^{(\theta -1)/\theta }+(1-\gamma )D^{(\theta -1)/\theta }\right]}^{\theta /(\theta -1)}$$

where θ is the constant elasticity of substitution between the domestic and traded goods (Armington elasticity), M is the trade volume which in this study is the volume of imported goods and D is the volume of domestic goods. γ and 1−γ are the distribution parameters associated with M and D (indices for industry groups are omitted).

Cost minimization subject to the above utility function implies the first-order condition that the marginal rate of substitution between M and D should equal the corresponding price ratio $\frac{P^{M}}{P^{D}}$. This condition can be solved for the quantity ratio of imported and domestic products in Equation (2) as follows:

(2) $$\frac{M}{D}={\left[(\frac{\gamma }{1-\gamma })\frac{P^{M}}{P^{D}}\right]}^{\theta }$$

where $P^{M}$ and $P^{D}$ are trade and domestic prices, respectively. Re-writing Equation (2) in logarithmic form, we have

(3) $$\ln\left[\frac{M}{D}\right]=\theta \ln\left[\frac{\gamma }{1-\gamma }\right]+\theta \ln\left[\frac{P^{M}}{P^{D}}\right]$$

Elasticities are unlikely to be equal across sectors [29]. A simplified form of the level relationship based on bilateral industry/sector data is as follows:

(4) $$\ln M_{ijt}^{UK}=\alpha_{0,ij}+\alpha_{1,ij}\ln\frac{P_{ijt}^{M}}{P_{ijt}^{D}}+\alpha_{2,ij}\ln D_{i,t}^{UK}+\varepsilon_{ijt}$$

where $\alpha_{1}$ is the price elasticity of substitution. Equation (4) can be estimated within a panel framework, which helps in testing the equivalence of coefficients across industries/sectors: $\alpha_{k,ij}=\alpha_{k,ij}\forall i$. Indeed, in a panel context we can also combine trade from a number of countries and all industries/sectors in our basic specification: $\alpha_{k,ij}=\alpha_{k,ij}\forall i,j$.

Similarly, the UK’s imports from China (j is China) can be written as:

(5) $$\ln M_{i,cht}^{UK}=\beta_{0,ij}+\beta_{1,ij}\ln\frac{P_{i,cht}^{M}}{P_{it}^{D}}+\beta_{2,ij}\ln D_{it}^{UK}+\varepsilon_{ijt}$$

where $M_{i,cht}^{UK}$ is the UK’s imports from China for sector i, $\frac{P_{i,cht}^{M}}{P_{ijt}^{D}}$ is the ratio of the prices of China to that of the UK in sector i.

Take Equations (4) and (5): the UK relative imports can be written thus:

(6) $$\ln\frac{M_{ijt}^{UK}}{M_{i,cht}^{UK}}=\gamma_{ij0}+\gamma_{1,ij}\ln\frac{P_{ijt}^{UK}}{P_{i.cht}^{UK}}+\varepsilon_{ijt}$$

where $\frac{M_{ijt}^{UK}}{M_{i,cht}^{UK}}$ is the ratio of the UK imports from country j to the UK imports from China in sector i, $\frac{P_{ijt}^{UK}}{P_{i.cht}^{UK}}$ is the ratio of the import price of the UK to that of China in sector i. As expected, when the import price from China is lower than the others, the relative demand from China will increase, so $\gamma_{1,ij}<0$.

In order to have the effects of political trade barriers reflected in the textile imports, three dummies were included in our specification, as follows,

(7) $$\begin{array}{cc} \ln\frac{M_{ijt}^{UK}}{M_{i,cht}^{UK}}=\gamma_{ij0} & +\gamma_{1,ij}\ln\frac{P_{ijt}^{UK}}{P_{i.cht}^{UK}}+\gamma_{2,,ij}D_{t}^{MFA}+\lambda_{3,ij}D_{t}^{WTO}\\ & +\lambda_{4,ij}D_{t}^{Tariff}+\lambda_{5,ij}D_{t}^{GBs}+\varepsilon_{ijt} \end{array}$$

where $D_{t}^{MFA}$ means that the Multi fibre Arrangement (MFA) was banned. In January 1995, the quota agreement in the MFA was banned by the ATC of the general trade agreement, which would have had an impact on China as an exporting country. $D_{t}^{MFA}$ is equal to 1 from 1995, and 0 before 1995.

$D_{t}^{WTO}$ means that China joined the WTO on 1 December 2000 and officially enjoyed the MFN treatment of GATT. Since then, Chinese goods have suffered injustice and quotas in international trade have been officially cancelled. The dummy variable from 2000 was 1, and before 2000 was 0.

$D_{t}^{Tariff}$ means that the export tariffs of 17 textile materials subject to quotas imposed by the EU were stopped in China. Since it is difficult to capture the change of tax [30], we set this factor as a dummy variable, which equals 1 from 2005 and 0 before 2005.

$D_{t}^{GBs}$ means a proxy of green trade barriers (GBs), namely Oeko-Tex Standard 100, which is the most representative ecological barrier in textile materials and products to trade and ensure that textile products are not harmful for the ecological environment and human health. Oeko-Tex Standard 100 was initially launched in 1992 and introduced into China in the 1990s. The dummy variable equals 1 from 1992 and 0 before 1992.

## 4. The Seemingly Unrelated Regression Model (SUR Model) and Data Description

We estimate nine equations across nine sectors of textile imports based on Equation (7). If the residuals across equations are uncorrelated, then ordinary least squares (OLS) would be an appropriate technique. However, if the residuals are correlated the equations could be linked. In the nine equations all of the variables, such as relative prices, have been used as regressors; thus the residuals of these equations are subject to cross correlation since they are also associated with various import demands for textiles across the same group of countries. 

The seemingly unrelated regression (SUR) model developed by Zellner (1962) [31] is a technique for analyzing a system of multiple equations with cross-equation parameter restrictions and correlated error terms. SUR is an extension of the linear regression model which allows correlated errors between equations. There are nine equations across the nine textile sectors. Each equation may satisfy the OLS assumptions, but the joint model which exhibits serial correlation due to the correlation of the error terms and OLS estimation will be inefficient. Using the SUR method to estimate the equations jointly, improves efficiency.

This paper focuses on analyzing the effects of Chinese textile raw materials on the UK imports from three trade regions: EU, ASEAN and North America. The countries included in these regions are listed in Table 7.

This analysis covers the bilateral imports of textile raw materials between the UK and other regions, namely the EEA, ASEAN, North America and China, Trade data in annual frequencies are taken from the OECD International Trade by Commodity Statistics-SITC Revision 3 (UN Comtrade), index 65, which was also categorized into 9 sectors and lists in Table 8, as follows.

This paper mainly investigates the role of China on the UK imports, especially the impact of relative import prices of the UK on its imports from three large economic trading areas including EEA, ASEAN and North America. Table 9 shows the data description of these two main variables in the three regions by a series of comparisons. It seems that compared with those of the EEA and North America, ASEAN exports to the UK were more severely affected by exports from China, which also implies that textile raw materials from ASEAN were not very competitive in the world market although it has obvious comparative advantages in its costs of labor, consistent with the results in Table 2.

## 5. Discussion

The set of results for the impact of relative price on sectoral textile relative imports is presented in Table 10 using the SUR model, where we consider the impact on sectoral trade from all regions and countries combined in a single panel. 

### 5.1. The Effects of Relative Imports Prices

The UK relative price of imports has a negative impact on imports from the three trade regions across all nine sectors and the magnitude effects vary with sectors and regions. In terms of trading with the EEA, UK imports of fabrics woven of man-made textile materials (sector 2), knitted or crocheted fabrics (sector 5) and floor coverings (sector 9) were more affected by Chinese textile products. When the relative price of UK imports from the EEA increased by 10%, UK relative imports in fabrics woven of man-made textile materials (sector 2) fell by 11.57% , knitted or crocheted fabrics (sector 5) fell by 10.96% and floor coverings (sector 9) fell by 12.31%. The impact of China on UK imports from the EEA was weaker when it came to products of textile yarn (sector 1) and cotton fabrics woven (sector 2). The magnitude of negative effects of Chinese textiles on UK imports became larger in the ASEAN and North America imports across all nine sectors with the exception of floor coverings (sector 9). Chinese textile raw materials, whether of technical-led man-made fabrics or cotton yarn related materials, had a significant impact on UK imports from the three trade regions, especially on the imports from ASEAN and North America. Since the UK textile imports were skewed to the EEA region, Chinese textile raw materials did not affect the UK demands from EEA too much, due to the trade protectionism in the EEA.

### 5.2. The Effects of International Trade Barriers

The effect of international trade barriers were also considered in the relative imports of the UK from the three trade regions. Before 1995, imports quotas from the developed countries were set by the MFA. Even though developing countries such as China, the ASEAN countries and Mexico have incomparable comparative advantage in textile materials production, the exports were limited by quotas. Table 10 shows that $D_{t}^{MFA}$ was not statistically significant in all the EEA’s sectors, but was positively significant on fabrics woven of man-made textile materials (sector 3) in ASEAN and North America and Tulle, lace, embroidery, ribbons etc. (sector 6) and made-up articles (sector 8) in ASEAN. This all implies that cancelling the quotas in the textile market has stimulated the high value added product exports of the labor intensive industries of developing countries. 

### 5.3. The Influence of China Joining WTO

After China joined the WTO in 2000 and began to enjoy most favored nation treatment, the comparative advantages in Chinese textile raw materials became overwhelming and have had a significant negative impact on the UK’s imports from all trade regions. In the EEA market, all textile sectors were negatively affected by export shocks from Chinese textile materials, especially for the categories of Knitted or crocheted fabrics (sector 5), Tulle, lace, embroidery, ribbons etc. (sector 6), and Special yarn and related products (sector 7), which implies the weaknesses of the EEA’s competitiveness in these sectors. The ASEAN market also confronted similar problems. Fabrics woven of man-made textile materials (sector 3) were mostly negatively affected by the Chinese textile materials shocks. However, it also shows that cotton fabrics (sector 2) and floor coverings (sectors), featured products of ASEAN countries, were not negatively influenced by China’s membership of the WTO and indicate that Chinese textile materials were not as competitive in the UK market as those of the ASEAN countries. Meanwhile, UK imports from North America market were hit by China’s exports, but the magnitude of negative effects became weaker than those in the ASEAN market. Among all sectors, special yarn and related products (sector 7) felt the greatest negative impact and cotton fabrics (sector 2) and all textile fabrics woven (sector 4) were not influenced at all.

### 5.4. The Effects of China’s Domestic Export Tax Policy

In this paper we also consider the Chinese policy of domestic tax on textile exports. After July 2005, when the export tax on 17 kinds of textile materials stops from China domestic side, we observe that $D_{t}^{Tariff}$ has significant impact on textile yarn (sector 1), fabrics woven of man-made textile materials (sector 3), knitted or crocheted fabrics (sector 5), tulle, lace, embroidery, ribbons etc. (sector 6) and special yarn and related products (sector 7). The effects were experienced in the EEA region on textile yarn (sector 1) and special yarn and related products (sector 7), other textile fabrics woven (sector 4) and tulle, lace, embroidery, ribbons etc. (sector 6) in North America. The more the UK importers demanded these products, the greater the comparative advantage of the Chinese textile materials, and the greater the negative effects on other trade regions.

### 5.5. The Environmental and Ecological Labelling in the UK Relative Raw Textile Imports

Oeko-Tex Standard 100 was adopted here to be a proxy of the green trade barriers (GBs) on textile products and related raw materials, which have significant and varied effects on the imports of textile raw materials from other trade regions. We assume that the GBs have adverse effects on imports. If so then when the magnitudes of the negative effects of GBs on these trade regions were greater than those on China/UK relative imports. For example, UK imports from the EEA were less affected by GBs except for fabrics woven of man-made textile materials (sector 3) and knitted or crocheted fabrics (sector 5), meaning that the restriction by the GBs may have more negative affects on the UK imports from the EEA of these two products than on those from China. As we found, the UK relative imports price shock in relation to China also hit these two sectors in EEA most severely, indicating that the GBs have raised the costs of production for these two products and reduced the UK’s relative imports. In the ASEAN region, GBs also had an adverse influence on the imports of textile yarn (sector 1), fabrics woven of man-made textile materials (sector 3) and other textile fabrics woven (sector 4). In the North America region, GBs negatively affected textile yarn (sector 1), fabrics woven of man-made textile materials (sector 3), knitted or crocheted fabrics (sector 5) and floor coverings (sector 9). Above all, with reference to China in the UK imports, GBs did have a negative impact on UK relative imports, especially on products with high value added features such as man-made textile materials from the EEA and North America, and floor coverings from the ASEAN and North America region.

With reference to the role of China, we investigate the UK imports from the EEA, ASEAN and North America by making comparisons. We find that textile price, product techniques, political trade barriers and export tax as well as green trade barriers all have a negative impact on the UK’s relative imports, especially of technical manual products and high value-added materials. China has comparative advantages in the international textile market but if it wants to maintain its sustainable competitiveness worldwide, it is still imperative to improve its comparative advantages in textile materials and guarantee its sustainable development in response to foreign demands. 

## 6. Conclusions

We investigate the role of China in the UK relative imports with EEA, ASEAN and North America and analyze the impact of factors including relative prices at each sector, China entry in WTO in 2000, cancellation of MFA in 1995 and China domestic tax policy in 2005 as well as environmental and ecological labeling starting in 1992 on the UK raw textile imports in relation to China. Compared with those of three trade regions in the world, some of China’s textile raw materials have stronger competitiveness and comparative advantages. The shocks of Chinese textile raw materials pricing on competitors in the UK markets are mainly reflected in highly value added textile raw materials, such as knitted or crocheted fabrics, which indicates that it is necessary to maintain and strengthen the characteristics of high value-added textile raw materials so as to enable foreign importers to recognize the originality of its products. Only when this comparative advantage is evident can the developed countries maintain the enthusiasm and sustainability of their imports of raw materials from China. 

China, having a labor-intensive industry with extensive supplies of labors, now has overwhelming comparative advantages in international competitiveness, which is a great challenge for the EEA, the ASEAN countries, the North American countries and other developing countries. With the reduction of political trade barriers and export taxes, Chinese textile raw materials with their high value added features have a generally negative impact on UK imports from other regions, but the impacts are insignificant or varied on the UK imports from the EEA, ASEAN and North America, with obvious advantages, which partly reflects the weakness of China’s textile raw materials in international competition, and prompts some suggestions for promoting the future development of China’s textile raw materials industry. After joining the WTO in 2000, Chinese textile exports increased sharply, but import requirements for textile raw materials for developed countries continued to strengthen from the perspective of environmental and ecological labeling. Although China’s textile raw materials still have a dominant position in the markets due to its technical manual high added value, China should also see the potential crisis of its own development, such as the lower competitiveness of its floor coverings in the ASEAN and North American markets. Improving the industry features of textile raw materials would be a channel for maintaining China’s sustainable development in the textile raw materials market.

The impact of China on UK imports from other regions may tend to become weaker regarding low value-added raw materials, and the products with more prominent advantages often contain higher technical composition and manual features. In the future, the rising prices of China’s labor and raw materials will lead to a price rise for high value-added products, which will cause a long-term negative impact on the international competitiveness of China’s textile raw materials. While maintaining its status regarding the export and processing of textile raw materials, China should continue to transform its design and innovation, comply with the historical trend and turn “made in China” into “created in China”. 

It would be valuable for further studies to be carried out seeking to investigate the UK relative imports in textile raw materials from more specific countries or regions, such as India and Bangladesh. Furthermore, it would be also worthwhile to consider more environmental factors in relation to textile raw materials or textile final products in further studies.

## Figures and Tables

**Table 1:** The amount and percentage of the UK textile raw materials imports from China and three free trade regions (unit: USD in millions).

Country/Region	1990		2000		2010		2016	
	Imports %		Imports %		Imports %		Imports %	
World	7017.65	100%	7265.29	100%	7163.52	100%	7217.23	100%
China	107.16	2%	373.79	5%	1379.87	19%	1456.72	20%
EEA	2954.15	42%	4010.39	55%	3298.82	46%	3237.83	45%
ASEAN	139.30	2%	323.30	4%	136.50	2%	118.40	2%
North America	417.64	6%	421.52	6%	293.05	4%	225.48	3%

Source: UN ComTrade SITC Rev. 3, 2017.

**Table 2:** Competition index in textile materials of China, EEA, ASEAN and North America in UK market (competition index = (X*_i_* − M*_i_*)/(X*_i_* + M*_i_*), Xi means country *i* exports to the UK, M*_i_* means country *i* imports from the UK).

Country/Region	1990	1995	2000	2005	2010	2016
China	-	0.691	0.693	0.796	0.829	0.850
EEA	0.203	0.255	0.256	0.266	0.277	0.240
ASEAN	0.419	0.496	0.455	0.192	0.112	−0.202
North America	−0.029	−0.009	−0.126	−0.177	0.029	−0.068

Source: UN ComTrade SITC Rev. 3, 2017.

**Table 3:** RCA in textile materials of China, EEA, ASEAN and North America in UK market (revealed comparative advantage index RCA*_i_* = (X*_i_*/X)/(X*_wi_*/X*_w_*), X*_i_*, X*_wi_* means textile export of country *i* to the UK and world, X, X*_w_* means total export of country *i* to the UK and the world. While RCA is calculated in this paper, added value is used instead of total export in order to avoid biases).

Country/Region	1990	1995	2000	2005	2010	2016
China	-	0.602	0.424	0.626	0.656	0.624
EEA	1.059	0.903	0.801	0.711	0.654	0.593
ASEAN	1.224	1.483	0.899	0.535	0.523	0.450
North America	1.132	1.003	0.552	0.502	0.483	0.438

Source: global value chain data base, 2017.

**Table 4:** Values of UK textile materials imports from EEA members (unit: dollars in millions).

**Year**	**Austria**	**Belgium**	**Bulgaria**	**Croatia**	**Cyprus**	**Czech**	**Denmark**	**Estonia**	**Finland**	**France**
1990	152.54	-	0.32	-	2.33	20.76	112.26	-	17.07	559.05
2000	82.38	853.54	1.91	3.07	0.61	-	62.55	29.14	8.37	485.63
2010	46.07	805.78	16.21	0.60	0.22	-	90.91	1.13	9.33	237.37
2016	48.72	730.26	19.70	0.17	0.07	-	95.85	0.24	16.70	224.99
**Year**	**Germany**	**Greece**	**Hungary**	**Iceland**	**Ireland**	**Italy**	**Latvia**	**Liechtentein**	**Lithuania**	**Luxembourg**
1990	-	57.54	6.76	1.80	305.98	755.63	-	-	-	-
2000	619.96	32.36	12.05	2.00	232.64	695.06	2.65	-	3.19	14.39
2010	578.79	22.21	32.55	0.06	85.43	527.41	3.31	-	4.82	29.41
2016	537.30	11.12	25.79	0.04	48.86	465.71	9.71	-	24.66	11.41
**Year**	**Malta**	**Norway**	**Holland**	**Poland**	**Portugal**	**Romania**	**Spain**	**Sweden**	**Slovenia**	**Slovakia**
1990	1.96	12.39	447.03	20.07	306.49	3.14	130.89	40.13	-	-
2000	0.29	8.43	365.68	34.69	219.53	4.35	185.13	33.45	11.18	6.16
2010	0.09	5.98	438.00	39.02	117.42	22.87	107.20	47.58	11.01	18.04
2016	0.03	4.39	499.25	84.71	127.14	19.14	151.54	40.91	5.77	33.65

Source: UN ComTrade SITC Rev. 3, 2017.

**Table 5:** Value of UK textile materials imports from ASEAN (unit: dollars in millions).

Year	Malaysia	Indonesia	Thailand	Philippines	Singapore	Brunei	Vietnam	Laos	Myanmar	Cambodia
1990	13.05	80.25	30.14	4.23	11.36	0.27	---	---	---	---
2000	37.67	169.22	99.15	7.51	1.26	0.94	7.28	0.06	---	0.18
2010	10.43	46.11	54.60	6.77	0.98	---	16.65	0.002	---	0.91
2016	6.27	26.55	33.77	5.94	0.73	0.008	38.21	0.03	---	6.93

Note: ASEAN members: Malaysia, Indonesia, Thailand, Philippines, Singapore, Brunei, Vietnam, Laos, Burma and Kampuchea. Source: UN ComTrade SITC Rev. 3, 2017.

**Table 6:** Value of UK textile materials import from North America (unit: dollars in millions).

Year	The U.S.	Canada	Mexico
1990	353.01	46.14	18.49
2000	397.34	16.70	7.48
2010	256.96	17.13	18.96
2016	201.85	10.81	12.83

Note: North America free trade region: the U.S., Canada, Mexico. Source: UN Com Trade SITC Rev. 3, 2017.

**Table 7:** Member countries in EEA, ASEAN and North America.

Region	Countries
EEA	Austria, Belgium, Bulgaria, Croatia, Cyprus, Czech, Denmark, Estonia, Finland, France, Germany, Greece, Hungary, Iceland, Ireland, Italy, Latvia, Liechtenstein, Lithuania, Luxemburg, Malta, Holland, Norway, Poland, Portugal, Romania, Spain, Sweden, Slovenia, Slovakia
ASEAN	Malaysia, Indonesia, Thailand, Philippines, Singapore, Brunei, Vietnam, Laos, Burma, Cambodia
North America	The United States, Canada, Mexico

**Table 8:** Sectors classification of index 65, textile raw materials.

Industry	Sectors
Textile yarn, fabrics, made-up articles and related products	(1) Textile yarn; (2) Cotton fabrics woven; (3) Fabrics woven of man-made textile materials, (4) Other textile fabrics woven; (5) Knitted or crocheted fabrics; (6) Tulle, lace, embroidery, ribbons, etc. (7) Special yarn and related products; (8) Made up articles; (9) Floor coverings.

Source: UN Comtrade SITC Rev. 3, 2017.

**Table 9:** Data description of Equation (7).

Sectors										
Variables		(1)	(2)	(3)	(4)	(5)	(6)	(7)	(8)	(9)
**EEA**										
$\ln\frac{M_{ijt}^{UK}}{M_{i,cht}^{UK}}$	Mean	−0.015	−1.631	−1.788	−0.930	−1.399	−1.38	0.738	−3.35	−1.778
	S.D.	2.549	2.366	3.375	2.011	2.969	3.286	2.070	2.155	2.740
$\ln\frac{P_{ijt}^{UK}}{P_{i.cht}^{UK}}$	Mean	0.139	0.990	1.023	−0.377	1.048	0.753	0.146	0.401	0.373
	S.D.	0.751	0.721	0.543	1.036	0.797	1.125	0.813	0.608	1.030
**ASEAN**										
$\ln\frac{M_{ijt}^{UK}}{M_{i,cht}^{UK}}$	Mean	−2.511	−3.85	−3.00	−4.844	−4.815	−3.628	−3.403	−4.502	−5.95
	S.D.	2.626	2.868	3.153	2.479	2.263	2.582	1.670	1.783	1.828
$\ln\frac{P_{ijt}^{UK}}{P_{i.cht}^{UK}}$	Mean	−0.140	0.505	0.263	0.602	0.595	0.619	−0.083	−0.138	1.121
	S.D.	0.769	0.799	0.805	1.13	0.881	1.133	0.744	0.540	1.480
**North America**										
$\ln\frac{M_{ijt}^{UK}}{M_{i,cht}^{UK}}$	Mean	−0.341	−1.489	−1.141	−0.980	−1.345	−1.432	−0.202	−1.931	−0.796
	S.D.	0.835	1.162	1.188	1.019	1.265	1.301	1.242	0.864	0.606
$\ln\frac{P_{ijt}^{UK}}{P_{i.cht}^{UK}}$	Mean	0.037	0.433	0.377	−0.082	0.469	0.381	0.082	0.236	−0.064
	S.D.	0.295	0.291	0.289	0.466	0.251	0.334	0.181	0.236	0.299

Source: UN Comtrade SITC Rev. 3, 2017.

**Table 10:** Impact of Chinese textile raw materials on UK imports using the SUR model.

	Variables	Three Trade Regions	Constant	$\ln\frac{P_{ijt}^{UK}}{P_{i.cht}^{UK}}$	$D_{t}^{\mathrm{MFA}}$	$D_{t}^{WTO}$	$D_{t}^{Tariff}$	$D_{t}^{GBs}$	*R*^2^	Obs
SITC										
**Sector 1.** Textile yarn		**EEA**	2.102 *** (6.32)	−0.769 ** (2.50)	−0.928 * (1.96)	−1.343 ** (2.38)	−1.472 ** (2.39)	0.386 (0.56)	0.390	152
		**ASEAN**	−2.448 *** (5.77)	−1.656 *** (6.47)	0.923 (1.75)	−1.320 *** (2.49)	0.750 (1.53)	−0.050 ** (2.12)	0.354	166
		**North American**	0.419 ** (2.00)	−0.886 *** (4.35)	0.225 (0.99)	−1.092 *** (4.34)	−0.646 *** (2.62)	−0.337 ** (2.19)	0.437	87
**Sector 2.** Cotton fabrics woven		**EEA**	0.578 *** (1.62)	−0.438 *** (6.78)	−0.526 (1.14)	−1.107 ** (2.14)	0.657 (1.31)	0.066 (0.09)	0.328	153
		**ASEAN**	−2.296 *** (4.76)	−1.604 *** (6.46)	0.201 (0.32)	−0.621 (1.00)	−1.045 ** (2.01)	1.105 (1.29)	0.322	149
		**North American**	−1.414 *** (4.09)	−0.624 ** (2.41)	0.118 (0.30)	−0.073 (0.17)	−0.439 (1.11)	0.274 (0.52)	0.412	81
**Sector 3.** Fabrics woven of man-made textile materials		**EEA**	2.547 *** (5.89)	−1.157 *** (3.09)	−2.227 (1.80)	−1.931 ** (2.95)	−1.348 ** (2.25)	−1.199 * (1.96)	0.478	153
		**ASEAN**	−1.208 (3.23)	−1.760 *** (8.79)	2.309 *** (4.48)	−2.190 *** (4.37)	−1.021 ** (2.43)	−0.668 ** (1.94)	0.592	164
		**North American**	−0.655 *** (2.66)	−1.288 * (1.79)	0.786 *** (2.51)	−1.021 *** (3.05)	0.064 (0.24)	−1.02 * (1.77)	0.433	86
**Sector 4.** Other textile fabrics woven		**EEA**	0.024 (0.09)	−0.351 ** (2.91)	0.244 (0.59)	−1.147 ** (2.31)	−0.423 (0.95)	−0.175 (0.760)	0.247	153
		**ASEAN**	−3.435 (7.62)	−0.183 ** (2.00)	−0.304 (0.47)	−1.332 ** (2.15)	−1.139 ** (2.34)	−1.435 ** (2.04)	0.149	150
		**North American**	−0.795 ** (2.88)	−0.474 ** (2.30)	0.146 (0.47)	−0.039 (0.11)	−0.612 ** (2.23)	0.405 (1.70)	0.268	86
**Sector 5.** Knitted or crocheted fabrics		**EEA**	1.329 ** (2.76)	−1.096 *** (3.88)	0.431 (1.15)	−1.925 *** (3.17)	−2.506 *** (4.54)	−1.299 * (1.94)	0.442	153
		**ASEAN**	−2.430 *** (7.85)	−1.256 *** (8.13)	−0.377 (0.87)	−1.142 ** (2.85)	−1.884 *** (5.61)	−1.48 ** (2.39)	0.524	150
		**North American**	−0.266 (0.84)	−1.384 ** (2.20)	−0.394 (1.15)	−0.980 *** (2.86)	−1.101 *** (3.79)	−0.308 ** (2.07)	0.439	84
**Sector 6.** Tulle, lace, embroidery, ribbons, etc.		**EEA**	1.181 *** (2.84)	−0.371 *** (3.06)	−0.954 (1.26)	−2.581 *** (3.99)	−1.201 (1.41)	−0.240 (0.55)	0.364	153
		**ASEAN**	−1.906 *** (5.85)	−0.717 *** (5.64)	1.491 *** (3.29)	−1.862 *** (4.27)	−1.092 (2.93)	−0.464 (1.45)	0.541	164
		**North American**	−0.564 (1.88)	−0.284 *** (2.79)	0.389 (1.06)	−1.16 (3.12)	−0.341 (1.08)	−0.763 (1.75)	0.379	85
**Sector 7.** Special yarn and related products		**EEA**	2.728 *** (13.09)	−0.407 ** (2.10)	−0.963 (1.34)	−2.468 *** (6.21)	−1.310 ** (0.33)	0.603 (1.25)	0.701	102
		**ASEAN**	−2.217 (8.67)	−0.599 *** (3.05)	−0.262 (0.76)	−0.995 *** (2.77)	−0.238 1.64	−0.69 (1.71)	0.198	89
		**North American**	0.567 *** (4.05)	−0.570 *** (4.05)	0.190 (1.03)	−1.838 *** (7.27)	−0.434 1.33	−0.667 (1.36)	0.664	44
**Sector 8.** Made up articles		**EEA**	−2.406 *** (5.15)	−0.279 ** (2.52)	−0.371 (1.19)	−1.908 *** (3.89)	−1.470 (0.24)	−0.393 (1.26)	0.311	153
		**ASEAN**	−4.036 *** (18.61)	−1.410 *** (8.35)	0.922 *** (3.34)	−1.168 *** (4.20)	−0.406 (1.74)	−0.513 (1.37)	0.609	169
		**North American**	−1.471 *** (7.42)	−0.688 *** (2.72)	−0.368 (1.32)	−0.816 *** (2.91)	−0.394 (1.71)	−0.473 (1.49)	0.246	87
**Sector 9.** Flooring coverings, etc.		**EEA**	−1.265 *** (2.99)	−1.231 ** (9.28)	−0.417 (0.81)	1.874 (0.86)	0.360 (0.51)	0.274 (3.05)	0.402	146
		**ASEAN**	−5.575 (17.05)	−0.021 ** (2.20)	−0.489 (1.08)	−0.106 (0.23)	−0.558 (1.38)	−1.580 ** (2.48)	0.023	149
		**North American**	−0.222 (1.38)	−0.038 *** (3.41)	0.118 (0.62)	−0.737 *** (3.23)	−0.468 *** (2.37)	−0.655 ** (2.74)	0.275	77

Note: * means significant at 10%, ** means significant at 5%, *** means significant at 1%. Source: UN Comtrade SITC Rev. 3, 2017.
